# To Fear Is to Gain? The Role of Fear Recognition in Risky Decision Making in TBI Patients and Healthy Controls

**DOI:** 10.1371/journal.pone.0166995

**Published:** 2016-11-21

**Authors:** Annemarie C. Visser-Keizer, Herma J. Westerhof-Evers, Marleen J. J. Gerritsen, Joukje van der Naalt, Jacoba M. Spikman

**Affiliations:** 1 University of Groningen, University Medical Center Groningen, Department of Neurology, Groningen, the Netherlands; 2 University of Groningen, University Medical Center Groningen, Center for Rehabilitation, Groningen, the Netherlands; 3 University of Groningen, Department of Clinical and Developmental Neuropsychology, Groningen, the Netherlands; Radboud Universiteit, NETHERLANDS

## Abstract

Fear is an important emotional reaction that guides decision making in situations of ambiguity or uncertainty. Both recognition of facial expressions of fear and decision making ability can be impaired after traumatic brain injury (TBI), in particular when the frontal lobe is damaged. So far, it has not been investigated how recognition of fear influences risk behavior in healthy subjects and TBI patients. The ability to recognize fear is thought to be related to the ability to experience fear and to use it as a warning signal to guide decision making. We hypothesized that a better ability to recognize fear would be related to a better regulation of risk behavior, with healthy controls outperforming TBI patients. To investigate this, 59 healthy subjects and 49 TBI patients were assessed with a test for emotion recognition (Facial Expression of Emotion: Stimuli and Tests) and a gambling task (Iowa Gambling Task (IGT)). The results showed that, regardless of post traumatic amnesia duration or the presence of frontal lesions, patients were more impaired than healthy controls on both fear recognition and decision making. In both groups, a significant relationship was found between better fear recognition, the development of an advantageous strategy across the IGT and less risk behavior in the last blocks of the IGT. Educational level moderated this relationship in the final block of the IGT. This study has important clinical implications, indicating that impaired decision making and risk behavior after TBI can be preceded by deficits in the processing of fear.

## Introduction

In social situations human information processing capacity is to a large extent taken up by the processing of emotional information that is crucial for making adequate decisions [1–3]. In situations of uncertainty or risk, fearful stimuli are processed fast, making quick and adaptive responses possible [4–6]. Fear can be defined as an immediate, emotional reaction to perceived stimuli in the environment that signal danger, threat or high risk. Subjectively, it can be experienced as a feeling of uncertainty or lack of control in a current situation [7,8]. Expressions of fear on other people’s faces can indicate the presence of an important, yet undetermined source of danger or threat within the environment. Meta-analyses in healthy subjects showed that the ability to recognize fear from faces is influenced by gender and age: females outperform males and older subjects perform poorer than younger subjects [9,10]. Furthermore, of all basic emotions, fearful facial expressions appeared the most difficult to identify [11]. A pending question is to which extent the ability to perceive fearful expressions of others is related to the ability to make adequate decisions in ambiguous or risky situations. This question, already interesting in healthy subjects, becomes even more salient for patients with traumatic brain injury (TBI) who are often impaired in recognizing emotions, among which fear, and have difficulty regulating behavior [11–17]. Both adequate processing of fear and adaptive regulation of behavior have been found to rely on intact functioning of the amygdala and prefrontal cortex [18–21]. After TBI, lesions in the prefrontal cortex are common, both focal and diffuse [22]. Focal prefrontal lesions have been related to impairments in emotion recognition [13,23]. Diffuse injury to the brain outside the prefrontal cortex has also been found to impair facial emotion perception abilities, probably due to the disconnection of critical brain areas [24–26].

Studies from different fields suggest that the experience of emotions is an important process linked to emotion perception. Theories of ‘embodied cognition’ state that facial emotion perception involves perceptual, somatovisceral, and motoric re-experiencing of the relevant emotion in one's self [27]. In a study with healthy subjects, the ability to recognize facial emotions of others was indeed related to the level of interoceptive sensitivity [28]. Furthermore, neuroimaging experiments show that similar brain circuits are activated when people observe emotions of others and when they feel these emotions themselves [29]. With regard to fear, a significant relationship between the ability to identify fearful faces and the subjective experience of fear has been found in different psychiatric patient groups [30–34]. Croker & McDonald found that in patients with TBI, poorer performance on an emotion matching task was related to a reduction of the ability to experience fear after brain injury [35]. Furthermore, emotion recognition abilities have been related to self- or proxy ratings of emotional and behavioural problems and to ratings of social integration after TBI [12,36].

A frequently used task to measure risky decision making is the Iowa Gambling Task (IGT) [37]. The IGT requires the subject to maximize profit on a loan of play money. Participants must choose from four decks of cards and each card selection leads to monetary gains, but may also lead to losses. Based on recurrent feedback, subjects have to learn which decks are ‘risky’ and ‘safe’ in the long run. Emotionally-based physiological signals, so called ‘gut feelings’, are thought to unconsciously influence decision making in the IGT, before explicit knowledge of the task becomes present [37,38]. The first trials of the IGT are thought to measure decision making under ambiguity, while in the later trials decisions are made under known risk [39,40]. Patients with TBI in general and with frontal lesions in particular, often continue choosing the decks which have greatest risk of losing money and fail to develop an advantageous strategy over time [16,37,41–45]. It has been hypothesized that these patients might be unresponsive for (future) negative consequences and are controlled by the immediate prospects of a card selection. Hence, their behavior is not guided by an emotional and bodily ‘gut feeling’ that warns for possible negative outcomes [37,38]. Although patients with TBI can be impaired in both emotion recognition and affective decision making, to date, no studies have focused on whether these two processes are related. Studies conducted in other patient groups, patients with Parkinson’s disease and individuals with abstinence after substance dependence, found significant positive correlations between overall emotional facial recognition and decision making in the IGT [46,47]. A study on the impact of emotional context congruency on decision making under ambiguity in healthy subjects found that in subjects in the congruent test condition, i.e. the presentation of fearful faces after losses in the IGT, advantageous choices improved across the IGT. This was in contrast to subjects in the incongruent test condition, i.e. fearful faces shown after rewards, in which decision making on the IGT did not improve [48].However, this study by Aite and colleagues used emotional congruency as a between-subjects factor, but did not address the relationship between the ability to identify fearful faces and choice behavior across the IGT. To date, it has not been investigated in healthy subjects how the ability to recognize fear is related to affective decision making, and to which extent there are differences with regard to sex and age. Previous studies found poorer performance on the IGT for female subjects and older subjects [49,50]. In the present study we want to investigate in both healthy participants and patients with moderate to severe TBI how the ability to recognize fear influences the ability to learn from negative and positive feedback in a gambling task, taking into account possible influences of age, gender and educational level. In patients, the influence of the severity of the TBI and the impact of frontal lesions are analyzed. First of all, we expect patients with TBI to be more impaired than healthy controls on both fear recognition and affective decision making. For both groups, we expect an advantageous effect: a better recognition of fear will be related to a better regulation of risk behavior. It is hypothesized that subjects with poor recognition of fear, indicating a decreased ability to experience fear, will be less able to use fear as a signal to avoid disadvantageous choices. Greater injury severity and frontal damage are expected to negatively influence performance. Furthermore, it is questioned whether the recognition of fear influences decision making under ambiguity or whether it is related to more deliberate risk behavior. To investigate this, fear recognition will be related to risk behavior in the different phases of the IGT. To investigate if the identification of fear is most important for decision making and risk behavior, first, fear recognition ability is related to the course of choices in the IGT together with the ability to recognize other basic emotions.

## Methods

### Ethics statement

The ethical committee of the University of Groningen approved of the collection of data of healthy volunteers. Assessment of TBI patients was part of the inclusion procedure for a study on behavioral sequelae after TBI, which was approved by the medical ethical committee of the University Medical Center Groningen, the Netherlands. All participants gave informed written consent prior to study inclusion for their information to be used for research, and were treated in accordance with the declaration of Helsinki.

### Participants

The patient group consisted of 49 moderate to severe TBI patients. At the time of assessment, all patients were outpatients who were seen in the sub-acute or chronic stage for clinical follow-up by the trauma neurologist or the rehabilitation physician of the University Medical Center Groningen. Clinicians referred patients for assessment of behavioural and social cognitive deficits.

TBI patients were included if they were free from neurological conditions other than TBI, psychiatric conditions and substance abuse pre- or post-injury. Mean age of the 49 patients was 44.6 years (SD = 13.5, range 20 to 68 years). The group consisted of 38 male and 11 female patients. Mean educational level was 5.1 (SD 1.0, range 3–7 on a 7-point scale ranging from 1) primary school only to 7) university level). There was no significant relationship between age and educational level of TBI patients (rho = -.05, p =.71). For 43 patients data were available concerning the duration of Post Traumatic Amnesia (PTA); the mean PTA duration was 25.9 days (SD 25.1), with a range from 1 to 112 days. For 25 patients Glasgow Coma Scale scores were available, ranging from 3 to 15, with a mean of 8.6 (SD 4.0). Of 42 patients imaging data (MRI or CT) with regard to frontal damage was available: 27 patients had visible lesions to the frontal lobe, while 15 patients did not have frontal lesions. Mean time since injury (TSI) was 104.8 months (SD 103.3), with a broad range of 4 to 402 months.

Normal healthy volunteers participated in the study, matched to patients based on age, gender and educational level. They were recruited through newspaper advertisement and from social networks of the investigators. They were free from alcohol or drug abuse, and had no psychiatric or neurological history. Data were collected from 59 participants, 37 men and 22 women. Mean age of the group was 43.5 years (SD 1.9, range 20 to 70 years). Mean educational level was 5.4 (SD 1.1, range 2–7). Age of control subjects appeared to be negatively related to their educational level (rho = -.31, p = p =.02). Patients and healthy controls did not differ significantly in age (t = -.31, p =.76), distribution of educational level (Kolgorov Smirnov Z = 1.2, p =.13) or distribution of gender (X^2^ = 2.8, p =.10).

### Measures

Decision making was assessed using the Iowa Gambling Task (IGT). The current Dutch computerized version of the IGT is an implementation based on the original description [37]. Participants are placed behind a computer screen showing 4 decks of cards. The starting amount of money in the gambling task is 2000 euro. Participants must draw a card from one of the 4 decks and each card selection leads to monetary gains, but may also lead to losses. Participants are instructed to make the highest profit as possible at the end of the task. The instructions also include a ‘hint’ about the nature of the decks and inform explicitly that ‘some decks are worse than others’ and that ‘by staying away from worst decks subjects can win’.

Deck A and B are ‘risky’ and will eventually lead to a loss of money and deck C and D are ‘safe’ and will lead to profit. The frequency and magnitude of gains and losses differ for each deck and must be learned by the participant during the task. See Table 1 for the exact payoff structure of IGT used in the present study. On the computer screen feedback is given after each draw and shows the total amount of loss (in red) and profit (in green), the total previous amount of money and the current total amount of money.

**Table 1:** Payoff scheme of the Iowa Gambling Task.

	Deck A	Deck B	Deck C	Deck D
**Amount of gain per trial**	€ 100	€ 100	€ 50	€ 50
**Possible amount of loss per trial**	€ 150 - € 350	€ 1250	€ 50	€ 250
**Gain/Loss frequency (10 trials)**	5:5	9:1	5:5	9:1
**Number of net losses (10 trials)**	5	1	5	1
**Long-term outcome (10 trials)**	- € 250	- € 250	€ 250	€ 250

Cards are drawn 100 times. For each 20 draws, the number of cards drawn from advantageous decks (C+D) are subtracted from the number of cards chosen from the disadvantageous decks (A+B) resulting in 5 net block scores.

Recognition of basic emotions (happiness, fear, surprise, disgust, anger, sadness) was measured using the Ekman 60 Faces Test from the Facial Expression of Emotion- Stimuli and Tests (FEEST) [4,51]. The score on the recognition of fear (range 0 to10) was the primary measure used in the present study.

### Statistical analyses

The course of performance in the IGT was analyzed using General Linear Model, Repeated Measures Analyses, with Difference contrast and Group as a Between-subjects variable. Box’ test was used to test the homogeneity of variance-covariance matrices assumption which was always met. When the assumption of sphericity was not met, a Greenhouse-Geisser correction was applied. When variances were equal, Bonferroni post-hoc procedure was used, with unequal variances Games-Howel post-hoc test was applied. When patients and healthy controls were compared, T-tests were used for parametric data and Mann-Whitney U tests for ordinal data. Pearson’s correlation was used for parametric data and Spearman’s rho for ordinal data. The influence of emotion recognition on IGT performance was analyzed with GLM Repeated Measures, with net block scores (CD-AB) and choices B as outcome measures. Fear, Anger, Sadness, Disgust, Happiness, Surprise, Age and Educational level were entered as covariates; Group and Gender were used as Between-subjects-factors. These analyses were subsequently performed with Fear as emotion recognition covariate together with the covariates age and educational level. Group and Gender were entered as Between-subjects-Factors. In analyzing the course of net block scores and the course of choices for deck B in TBI patients, duration of PTA and TSI were entered as covariates, while the presence/absence of frontal lesions was entered as Between-subjects-factor. Alpha levels were two-tailed with p <. 05. All statistical analyses were performed with IBM SPSS Statistics 20.

## Results

### Decision making on the IGT

Table 2 shows means and standard deviations for the total number of draws for each deck for TBI patients and healthy controls. Calculated across the 100 trials of the IGT, healthy controls most often chose deck D, followed by deck B, deck C and deck A. Patients most often choose deck B, followed by deck D, deck C and deck A. Patients and controls only differed significantly in their choices for deck B (t = -3.0, p =.004). Patients with and without frontal lesions did not differ in their total number of choices for deck B (t =.19, p =.85). PTA duration was not significantly related to the total number of choices for deck B (r =.17, p =.26). TSI was not related to the total number of choices for deck B (r =.06, p =.69). Healthy controls had earned a mean of 133 euros at the end of the IGT (ranging from -1850 loss to +2150 profit), while TBI patients had lost a mean of 290 euros (ranging from -2800 loss to +1450 profit), a significant difference (t = 2,4, p =.02). In healthy controls higher age was negatively related to profit on the IGT (r = -.37, p =.004), while age was not significantly related to profit on the IGT in TBI patients (r = -.04, p =.77). In both healthy controls and TBI patients, males and females did not differ significantly in their profit on the IGT (controls: t = -.66, p =.51; patients: t = -.10, p =.92). Educational level had a small and non-significant positive relationship with profit on the IGT in both groups (controls rho =.25, p =.06, TBI rho =.23, p =.11). PTA duration was not significantly related to profit on the IGT in TBI patients (r = -.17, p =.27). TSI was not significantly related to profit on the IGT (r = -23, p =.13). TBI patients with and without frontal lesions did not differ significantly in profit on the IGT (t = -.06, p =.95).

**Table 2:** Mean (SD) total IGT deck draws and final IGT profit for TBI patients (n = 49) and healthy controls (n = 59).

	Deck A	Deck B	Deck C	Deck D	Final amount
**Controls**	14.9 (7.7)	29.3 (14.1)	25.2 (19.6)	30.6 (18.1)	2133 (901)
**Patients**	14.3 (6.9)	37.8 (16.0)	21.8 (14.6)	26.0 (14.1)	1710 (946)

Repeated measure analysis on the 5 net block scores (C+D—A+B) of the IGT showed an overall increase of positive net block scores from block 1 to block 5 (F (3.5, 366.5) = 3.2, p =.02) for both patients and healthy controls (Block*group, F(3.5, 366.5) = 0.5, p =.74). Healthy controls and TBI patients differed significantly in their performance on the IGT (F = (1, 106) = 5.4, p =.02). Healthy controls made more advantageous choices than TBI patients in Block 3 (F(1,106) = 3.9, p =.05), while this difference just did not reach significance in block 5 (F(1,106) = 3.5, p =.07).

Age appeared to influence the course of net block scores in both patients and controls (Block*age, F(3.5,362) = 2.6, p =.05; Block*age*group, F(3.5, 362) =.71, p =.57). Educational level or gender did not significantly interact with course of net block scores (Block*education, F(3.4,358) = 1.6, p =.17; Block*gender, F(3.4, 358) = 1.9, p =.12) in both healthy controls and TBI patients (Block*gender*group, F(3.5, 345) = 1.2, p =.29), Block*education*group, F(3.5, 345) =.32, p =.84).

**Fig 1:**
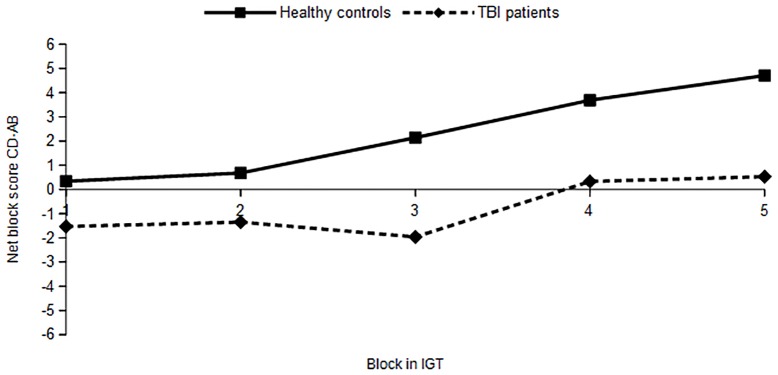
Course of net block scores in the IGT for healthy controls (n = 59) and TBI patients (n = 49).

In TBI patients, PTA duration (Block*PTA, F (3,121) =.34, p =.79), TSI (Block*TSI, F(3,133) =.46, p =.71) and the presence of frontal lesions (Block* Front, F(3,119) =.10, p =.96) did not affect the course of net scores from block 1 to block 5. See Fig 1 for the course of net block scores of healthy controls and TBI patients on the IGT.

### Emotion recognition

Table 3 shows mean FEEST emotion recognition scores of healthy controls and TBI patients, with healthy controls having significantly higher scores than patients on fear recognition (t = 2.0, p =.05), anger recognition (t = 3.0, p =.003) and total FEEST score (t = 2.6, p =.009).

**Table 3:** Mean (SD) emotion recognition scores for the six basic emotions and the total score of the FEEST for healthy controls (n = 59) and TBI patients (n = 49).

	Happiness	Fear	Disgust	Anger	Surprise	Sadness	Total
**Controls**	9.8 (.50)	6.4 (2.4)	7.2 (2.1)	8.1 (1.8)	9.0 (1.0)	7.0 (1.8)	47.5 (5.6)
**Patients**	9.7 (.74)	5.5 (2.6)	6.8 (2.4)	7.0 (2.1)	8.7 (1.4)	6.4 (2.1)	44.2 (7.3)

In healthy controls, women had significantly higher scores on fear recognition than men (women M = 7.3, SD = 2.1, range 3–10, men M = 5.9, SD = 2.5, range 1–10, t = -2.2, p =.03). Healthy females were significantly better than healthy males in recognizing anger and disgust (Anger: women M = 8.9, SD = 1.5, men M = 7.7, SD = 1.9, t = -2.5, p =.02; Disgust: women M = 8.0, SD = 2.0, men M = 6.8, SD = 2.0, t = -2.2, p =.03). In addition, total FEEST scores were significantly higher in healthy females than in healthy males (women M = 50.6, SD = 4.5, men M = 45.7, SD = 5.5, t = -3.5, p =.001).

In healthy controls, higher educational level was significantly related to higher fear recognition (rho =.26, p =.048), higher anger recognition (rho =.37, p =.004), higher recognition of disgust (rho =.30, p =.02) and higher total FEEST score (rho =.44, p =.000). Age was significantly related to total FEEST score, with higher age related to poorer emotion recognition (r = -.26, p =.05).

In TBI patients, there were no significant differences between men and women in the recognition of the six basic emotions. Age of patients was not significantly related to emotion recognition. Higher educational level was significantly related to higher fear recognition ability (rho =.37, p =.01) and to poorer recognition of surprise (rho = -.35, p =.02). PTA duration was not significantly related to emotion recognition ability. TSI was not significantly related to emotion recognition, except for a significant positive correlation between TSI and recognition of anger (r =.38, p = 01). Mean emotion recognition scores of patients with (n = 27) and without (n = 15) frontal lesions did not differ significantly.

### Emotion recognition and advantageous decision making

Repeated measure analysis on the influence of emotion recognition ability on the course of net block performance (A+B—C+D) in the IGT showed that fear recognition was significantly related to the course of decision making in the IGT (Block* Fear F (3.5, 337) = 3.2, p =.02). Recognition of anger (Block*Anger F (3.5, 337) =.69, p =.58), disgust (Block* Disgust F (3.5, 337) =.78, p =.52), happiness (Block*Happiness F (3.5, 337) = 1.2, p =.33), sadness (Block*Sadness F(3.5, 337) =.44, p =.75), and surprise (Block*Surprise F (3.5, 337) =.72, p =.56) were not significantly related to the course of decision making on the IGT. Three way interactions with block, emotions and group were not significant, indicating no differences in the influence of emotion recognition on the course of decision making for healthy controls and TBI patients. Three way interactions with demographical variables (gender, age and educational level), block and emotions showed no significant effects,

### Fear recognition and advantageous decision making

Both fear recognition and decision making were significantly worse in TBI patients compared to healthy controls. In healthy controls, but not in TBI patients, fear recognition correlated significantly with total profit on the IGT (controls, r =.31, p =.02, TBI, r =.16, p =.27).

Fear recognition scores were related to the course of net block performance (A+B—C+D) in the IGT for both healthy controls and TBI patients. GLM repeated measure analyses showed a main effect of block (F (3.5, 308) = 3.3, p =.01), an interaction between fear recognition and the course of net block score (Block*Fear F(3.5, 308) = 5.9, p =.00) but no interaction between course, fear and group (Block*Fear*Group F(3.5, 308) = 1.1, p =.38). Thus, fear recognition ability significantly interacted with the course of choice behavior in the IGT in healthy controls and patients. Post-hoc contrast showed the interaction between fear and choice behavior in the IGT to be significant in block 4 (Block 4 vs previous, Block*Fear, F(1,104) = 4.3, p =.04) and block 5 (Block 5 vs previous, Block*Fear, F(1,104) = 15.2, p =.000). This effect was similar for both groups (Block 4 vs previous, Block*Fear*Group, F(1,104) =.05, p =.83; Block5 vs previous, Block*Fear*Group, F(1,104) = 2.1, p =.15). Higher fear recognition ability was related to more advantageous choices in the last blocks of the IGT in both healthy controls and TBI patients.

Age was significantly related to the course of net block scores in the IGT for patients and controls. Age, however, did not interact significantly between fear recognition and net block scores in the IGT (Block*Fear*Age, F(3.6, 368) = 2.0, p =.10). Educational level was positively related to fear recognition ability in patients. Educational level also interacted with fear recognition and net block scores in the IGT (F(3.6,366) = 2.8,p =.03), which appeared to be similar for patients and controls. Contrasts showed that this interaction effect was present in the last block of IGT (Block 5 vs previous, Block*Fear*Education, F(1,102) = 4.6, p =.04). When age was also entered into this model, it did not appear to significantly moderate the relationship between educational level and fear recognition (Block*Fear*Education*Age, F(3.6,363) = 2.0, p =.10).

Fear recognition ability was significantly better in female compared to male controls. Gender, however, did not interact significantly between fear recognition and course of IGT net block scores (F(3.5, 365) = 2.1, p =.09). This was found in patients and healthy controls (Block*group*gender*fear F(10.5, 347) = 1,5, p =.13).

### Fear recognition and risk behavior

**Fig 2:**
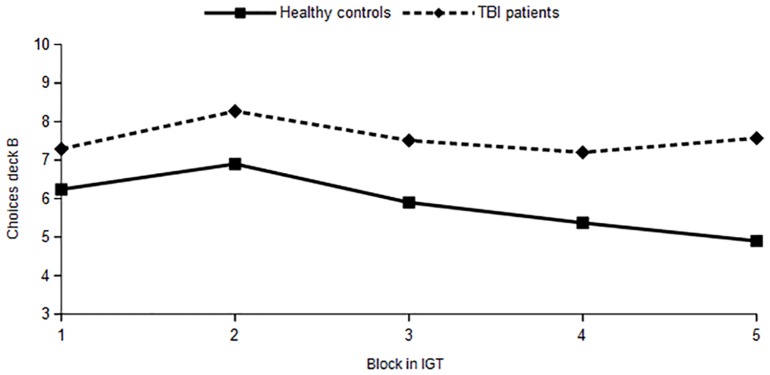
Course of number of choices for risky deck B in the IGT for healthy controls (n = 59) and TBI patients (n = 49).

With regard to risky choices, healthy controls and TBI patients differed significantly in the amount of total choices for deck B. See Fig 2 for the course of choices of Deck B of the IGT for both patients and controls. GLM Repeated measure analysis showed no overall effect of block (Block, F(3.5, 374) = 2.1, p =.09) a significant effect of group (F (1, 106) = 8.7, p =.004), with no significant difference for the course of choices for deck B between patients and controls (Block*group, F(3.5, 374) =.68, p =.59). TBI patients did significantly chose deck B more often than healthy controls in block 4 (t = -2.1, p =.04) and block 5 (t = -2.7, p = 008).

Repeated measure analysis on the influence of emotion recognition ability on the course of choices for deck B in the IGT showed that only fear recognition was significantly related to the course of choices of deck B (Block* Fear F (3.6, 346) = 4.5, p =.002). Recognition of anger (Block*Anger F (3.6, 346) = 1.6, p =.19), disgust (Block* Disgust F (3.6, 346) =.45, p =.76), happiness (Block*Happiness F (3.6, 346) =.78, p =.53), sadness (Block*Sadness F(3.6, 346) =.74, p =.55), and surprise (Block*Surprise F (3.6, 346) =.28, p =.88) were not significantly related to the course of choices for deck B on the IGT. Three way interactions with block, separate emotions and group were not significant, indicating no differences in the influence of emotion recognition on the course of choices for deck B for healthy controls and TBI patients.

Analyzed as a separate emotion, fear recognition ability significantly interacted with the course of choice behavior of deck B in both patients and controls (Block*Fear, F(3.7, 380) = 7.3, p =.000; Block*Fear*Group, F(3.7,380) =.54, p =.69). Post-hoc contrast showed that fear recognition interacted with choosing deck B in block 3 (Block 3 vs previous, Block*Fear, F(1,104) = 12.8, p =.001) and block 5 of the IGT (Block 5 vs previous, Block*Fear, F(1,104) = 13.9, p =.000). This interaction was similar in patients and healthy controls. Further analyses showed a negative correlation in both groups, with higher fear recognition abilities related to choosing deck B less often (Block 3, Controls: r = -.27, p =.03, TBI: r = -.32, p =.02; Block 5, Controls: r = -.57, p =.00, TBI: r = -.31, p =.03).

Age did not interact significantly with the course of choices for deck B (Block*age, F(96,156) = 1.3, p =.10), nor with fear recognition and the course of choices for deck B (Block*fear*age, F(96,156) = 1.1, p =.33). Educational level interacted with the course of choices for deck B (Block*Education, F(3.7, 370) = 2.5, p =.05), but there was no significant three-way-interaction between educational level, fear recognition and the course of choices for deck B (Block*Fear*Education, F(3.7,370) = 2.1, p =.09). Gender was neither related to the course of choices for deck B (Block*Gender, F(3.6,371) = 1.4, p =.25), nor did it show an interaction between fear recognition and course of choices for deck B (Block*Fear*Gender, F(3.6,371) = 1.2, p =.32).

In TBI patients, the presence of frontal lesions was neither related to the course of choices for deck B (Block*Lesion, F(3.0,123,5) = 0.1, p =.95) nor did it show an interaction between fear recognition and choices for deck B (Block*Fear*Lesion, F(3.0,117.9) =.11, p =.95). PTA-duration was not related to the course of choices for deck B (Block*PTA, F(3,124) =.29, p =.83). Also, PTA-duration did not interact with fear recognition ability in influencing the course of choices for deck B (Block*Fear*PTA, F(3,121) = 1.4, p =.25). TSI was neither related to the course of choices for deck B (Block*TSI, F(2.9,99) =.62, p =.60) nor did it interact with fear recognition ability in influencing the course of choices for deck B (Block*Fear*TSI, F(2.9, 95) =.81, p =.49).

## Discussion

This is the first study that investigated and indeed found a relation between facial fear recognition ability and gambling behavior in both healthy subjects and patients with moderate to severe TBI. In both groups, a higher ability to recognize fear, but not a higher ability to recognize other basic emotions, was related to the development of a more advantageous strategy and to the course of risky choices across blocks in the IGT. As expected, patients with TBI were significantly more impaired in recognizing fear from faces than healthy subjects [13–15]. Moreover, TBI patients on average lost money on the IGT, while healthy subjects made profit. Regardless of injury severity, almost 80% of the TBI patients appeared not to develop a preference for profitable decks (net block score > 10), which is very similar to other studies in TBI patients [16,41,45,52,53].

In both TBI patients and healthy controls decision making on the IGT was related to the ability to perceive fear. This relationship between perception and action is thought to be mediated by the ability to subjectively experience fear [27,28]. A recent study showed that poorer facial affect recognition and higher alexithymia, the ability to identify and describe feelings, were indeed related after TBI [54]. One of the mechanisms underlying subjects’ ability to experience emotions of others is mimicry [55,56]. Similar brain regions are involved in the detection of emotional facial expressions and in the activation of facial muscles necessary for the expression of that specific emotion [57]. Furthermore, experimentally blocking facial mimicry in healthy subjects can selectively impair the recognition of an emotional expression [58]. After TBI, patients can show a reduced mimicry of facial expressions, particularly for negatively valenced emotions [59,60]. In healthy subjects, females exhibit greater facial mimicry than males when viewing expressions of others [61,62]. Decision making guided by experienced emotions will be preceded by important processes like attending to and identification of emotional signals [63]. Greater interoceptive ability, as measured by more adequate attention to cardiac signals, has been related to better decision making on the IGT in healthy subjects [64,65]. Furthermore, the ability to experience feelings more intensely was related to higher decision making performance in a stock investment simulation task. In this experiment, healthy subjects who were better able to differentiate negative feelings achieved higher decision making performance, which was mediated by an enhanced ability to regulate the influence of their emotions on decision making [63]. However, the specific impact of the experience of fear on decision making was not examined in this stock investment study. In the present study, we found recognition of fear, and not the recognition of other basic emotions, to be associated with overall decision making and with risk behavior on the IGT. According to Zeelenberg and colleagues (2008) each specific emotion has its own motivational goal, creating a unique effect on behavior. In line with the present results, they state that the avoidance of danger or risk is the motivational goal of fear [66].

In the present study, we also investigated in TBI patients and healthy controls whether fear recognition influences decision making under ambiguity or whether it is related to more deliberate behavior. Maia and McClelland (2004) identified three levels of awareness in the IGT [40]. At the lowest level (0) participants have no conscious knowledge about the profit or loss of decks (block 1). At Level 1, participants do show a conscious preference for advantageous decks, but do not have explicit knowledge about the pay-off structure (block 2). At Level 2, participants have gained this knowledge and can use it to explain their preference for the good decks. Most explicit awareness is present at the end of the task, in block 5. Our findings show that in both healthy controls and TBI patients better fear recognition is significantly related to advantageous choices in the last two blocks of the IGT, indicating that fear can be consciously used as a signal to choose advantageously. This ability appeared superior for those patients and controls who were more highly educated. This suggests that affective and cognitive abilities interact in influencing deliberate decision making behavior [67]. Evans (2011) describes the dual-process theory of reasoning in which two distinct types of reasoning compete in decision making: an intuitive-heuristic form of reasoning, independent from working memory or cognitive ability and a slower, executive-analytic from of reasoning that does heavily depend on cognitive ability [68]. Both abilities appear necessary to gain insight in and to learn the pay-off structure in the IGT.

In the current study, fear recognition was not only related to overall decision making in both groups, but in particular to risky choice behavior at the end of the IGT. In both TBI patients and control subjects we found that fear was explicitly used as a warning signal not to choose disadvantageously. Heilman and colleagues (2010) also found in healthy subjects that explicit use of fear was related to risk behavior in the IGT [69]. In the study by Heilman, subjects were asked to reappraise fear. When subjects reduced the experience of this negative emotion, they subsequently showed more risk taking in the IGT. In our study, the relationship between fear and choosing risky deck B was significant in block 3 and block 5 of the IGT. This suggests that, in line with Maia and McClelland’s model, emergent knowledge with regard to the risk of losing money is tested, before it is used explicitly [40]. This also fits the dual process theory of reasoning, in which intuitive and analytic reasoning interact [68].

As expected, we found that TBI patients performed more poorly on the IGT than healthy controls. Originally, the IGT was developed to assess deficits in affective decision making after ventromedial prefrontal cortex (VMPFC) damage [38]. However, we found no effect of the presence of frontal lesions on the course of net block performance, which is in line with several studies that found impaired performance on the IGT after TBI in general, implying that this is not exclusively limited to patients with frontal damage [16,41,42,70]. Frontal processes can be impaired without focal frontal lesions, by the disruption of underlying networks [71].

In both healthy controls and TBI patients, a negative effect of age on net block performance on the IGT was found. Performance on the IGT is based on localized but also on distributed mental information processes such as memory functioning, which are sensitive to age [16,50,72]. We did not find gender to affect the course of net block scores, although in previous studies in healthy subjects a female disadvantage on the IGT has been described [49,73]. Similar to previous studies, we found healthy females to be better in recognizing fear than healthy males [9]. In both male and female healthy controls, the ability to recognize fear was significantly related to the amount of profit on the IGT.

According to Yechiam and colleagues (2005) different psychological components can underlie poor performance on the IGT, including a motivational tendency to attend to gains, a learning process to attend to recent outcomes and a response tendency with poor consistency of choices [52]. With regard to deck choices in the IGT, we found that TBI patients only differed from healthy subjects in choosing risky deck B more often, a difference that became significant at the end of the IGT. Deck B results in the greatest losses, but can also yield the highest immediate rewards, which might attract the attention of patients. The course of choices in the IGT, however, did not differ between patients and controls. This suggests that TBI patients do show some learning in the IGT, but have a delayed learning rate compared to healthy controls. Studies in healthy subjects showed that learning on the IGT is not completed after 100 trials and performance on the IGT can be enhanced with the addition of extra trials [74,75]. If this is the case for healthy controls, probably TBI patients would also benefit from more trials in the IGT. Bonatti and colleagues (2008) suggest that optimal learning in the IGT is based on both flexibility and stability of choices. In their study, TBI patients showed reduced flexibility and stability in their choices, reflecting the influence of executive functioning on performance on the IGT [45]. In the current study, higher fear recognition was related to less risk behavior in TBI patients in block 3 of the IGT. Nevertheless, they showed greater risk taking than healthy controls in the following blocks. This suggests that TBI patients were less able to consistently guide their behavior. The current results show that poorer recognition of fear is one of the underlying factors.

The present study design has some limitations. Fear recognition and risk behavior were measured, with the assumption that a better identification, experience and regulation of fear underlies behavior. It would have been valuable to additionally measure the ability of subjects to experience and appraise fear, either by questionnaires or by physiological measures. This might also give more insight into the specific deficits in the experience of fear in TBI patients. The advantage of the inclusion of an emotion recognition task is, however, that it allows objective measurement. Different from questionnaires, its responses do not depend on patients’ insight, which can also be impaired after TBI. The patient group used in the current study is a selection of patients with moderate to severe injury and behavioral difficulties. It is important to also analyze this relationship in a larger and more heterogeneous sample of TBI patients. The majority of the TBI patients in this study was male, and the inclusion of more female patients is preferred. In a greater sample of male and female patients, gender differences with regard to the influence of fear recognition on decision making can be analyzed in greater detail and with more power. However, male dominance is a characteristic of the TBI population [76].

In conclusion, the most important finding of the present study is that fear recognition is related to deliberate choice and risk behavior in both healthy controls and TBI patients suggesting strongly that deficits in behavior are preceded by deficits in emotion processing. This is a clinically relevant finding because deficits in emotion processing might be the target of research and treatment for TBI patients with risky and inappropriate behavior. So far, only few studies have investigated the effect of training of underlying deficits in emotion processing in TBI patients [15,77,78]. A recently conducted randomized placebo-controlled trial showed that, even in chronic TBI, facial affect recognition skills can be improved [78]. In line with theories on the motivational function of specific emotions, the current study suggests that it is valuable to extent research beyond the valence of emotions and to focus on the impact of specific emotions on specific behavior [63]. In clinical practice, deficits in emotion processing, especially in the processing of fear, should be taken into account in TBI patients with difficulty regulating their behavior in complex situations.
